# Aortic regurgitation and aortic valve replacement during repair of acute aortic dissection

**DOI:** 10.1186/1749-8090-8-S1-O44

**Published:** 2013-09-11

**Authors:** T Yamamoto, T Takano, T Terasaki, Y Wada, T Seto, J Amano

**Affiliations:** 1 Department of Cardiovascular Surgery, Shinshu University School of Medicine, Matsumoto, Japan

## Background

It is still on debate whether an aortic valve should be preserved or repaired or replaced during surgery for AAD although aortic regurgitation (AR) with acute aortic dissection (AAD) is recently treated with valve-sparing root repair.

## Methods

This study included 51 patients who had AR more than degree II before operation out of 271 patients who underwent surgery for AAD from January 2013 to May 2005. We performed repair of AAD without aortic valve surgery in 36 patients (group P) whereas repair with aortic valve replacement in 15 patients (group R). In-hospital mortality morbidity, mid-term mortality, major cardiovascular and neurological event was retrospectively obtained and compared between 2 groups.

## Results

Age was 67 ± 11.3 and 57 ± 14.7 years old in group R and P, respectively (p=0.611). Preoperative mean AR degree was 2.67 ± 0.74 in group P and 2.77 ± 1.1 in group R (p=0.735). Mean follow-up in group P was 20.9 ± 19.3 months and was 35.6 ± 26.9 months in group R.

In-hospital mortality occurred in each patient of group P and R (p=0.814). Cardiopulmonary bypass time was significantly shorter at 297 ± 72.0 min in group P than group R of 424 ± 145.6 min (p=0.004). Postoperative complications occurred in 11.1% of group P, 33% of group R (p=0.058). Late death was 8.5% in group P, and14% in group R (p=0.55). Cerebrovascular events occurred in 2.9% of group P, and 13.3 % of group R (p=0.192). Cardiovascular event was observed in 5.7% of group P, and in 14.3% of group R (p=0.567). In group P, AR was decreased to 0.77 degree immediately after the surgery and maintained at 1.16 degree 20.9 months after the surgery. No patient received aortic valve surgery in group P during follow-up although one patient required re-valve replacement in group R.

## Conclusion

AAD repair without aortic valve replacement could be applicable when the patients presented with II –III degree of AR to reduce operation time and possible morbidity.

